# Probiotics *Bacillus cereus* and *B. subtilis* reshape the intestinal microbiota of Pengze crucian carp (*Carassius auratus* var. Pengze) fed with high plant protein diets

**DOI:** 10.3389/fnut.2022.1027641

**Published:** 2022-10-19

**Authors:** Jiamin Li, Peng Fang, Xinwen Yi, Vikas Kumar, Mo Peng

**Affiliations:** ^1^School of Animal Science and Technology, Jiangxi Agricultural University, Nanchang, China; ^2^Shenzhen Aohua Group Co., Ltd., Shenzhen, China; ^3^Department of Animal, Veterinary and Food Sciences, Aquaculture Research Institute, University of Idaho, Moscow, ID, United States

**Keywords:** plant protein, Pengze crucian carp, intestinal microbiota, ecological network, microbial function

## Abstract

The intestinal dysfunction induced by high plant protein diets is frequently observed in farmed fish, and probiotics of *Bacillus* genus were documented to benefit the intestinal health through the modulation of intestinal microbiota without clearness in its underlying mechanism yet. Fusobacteria, Proteobacteria, and Firmicutes were observed to be the dominate phyla, but their proportion differentiated in the intestinal bacterial community of Pengze crucian carp (*Carassius auratus* var. Pengze) fed different diets in this study. Dietary supplementation of *B. cereus and B. subtilis* could reshape the intestinal bacterial community altered by high plant protein diets through a notable reduction in opportunistic pathogen *Aeromonas* together with an increase in *Romboutsia* and/or *Clostridium_sensu_stricto* from Firmicutes. Due to the alteration in the composition of bacterial community, Pengze crucian carp exhibited characteristic ecological networks dominated by cooperative interactions. Nevertheless, the increase in *Aeromonas* intensified the competition within bacterial communities and reduced the number of specialists within ecological network, contributing to the microbial dysbiosis induced by high plant protein diets. Two probiotics diets promoted the cooperation within the intestinal bacterial community and increased the number of specialists preferred to module hubs, and then further improved the homeostasis of the intestinal microbiota. Microbial dysbiosis lead to microbial dysfunction, and microbial lipopolysaccharide biosynthesis was observed to be elevated in high plant protein diets due to the increase in *Aeromonas*, gram-negative microbe. Probiotics *B. cereus and B. subtilis* restored the microbial function by elevating their amino acid and carbohydrate metabolism together with the promotion in the synthesis of primary and secondary bile acids. These results suggested that dietary supplementation of probiotics *B. cereus and B. subtilis* could restore the homeostasis and functions of intestinal microbiota in Pengze crucian carp fed high plant protein diets.

## Introduction

Aquaculture industry, one of the fastest growing animal food-producing sectors in agriculture, provides a large amount of high-quality protein to consumers around the world (1, 2). In recent decades, intestinal dysfunction is widely observed in farmed fish due to the excessive use of plant protein in commercial feed, and substantial evidences indicated that the occurrence of intestinal dysfunction is usually accompanied by a dysbiosis of intestinal microbiota (3–5). It is well-accepted that micro-ecological ecosystem formed by symbiotic microbes plays a crucial role in the health status of host through production of digestion-related enzymes, vitamins synthesis, protection from pathogens, and promotion of immune maturation, etc. (6, 7). Hence, regulating the homeostasis of intestinal microbiota would be one effective strategy to maintain the health of farmed fish.

Probiotics, defined as live microorganisms, have attracted more and more attentions for their benefits to the homeostasis of intestinal microbiota in aquatic animal, as well as growth performance, nutrient digestion, antioxidant capacity, and immunity system (8). *Bacillus* genus serves as one of the most common probiotic species and is widely applied to aquaculture industry (9, 10). Numerous researches have investigated the effect of *Bacillus* species on the intestinal microbiota of fishes like grass carp (*Ctenopharyngodon idellus*) (11), turbot (*Scophthalmus maximus*) (12), common carp (*Cyprinus carpio* L.) (13), olive flounder (*Paralichthys olivaceus*) (14), Nile tilapia (*Oreochromis niloticus*) (15), and tongue sole (*Cynoglossus semilaevis*) (16). Nevertheless, most of these studies in farmedc fish focus on the effects of *Bacillus* species on the composition of bacterial community, which is not enough to interpret the modulation of intestinal microbiota homeostasis by probiotics.

The number of species and their abundance are the most basic elements for constructing the bacterial community, whereas the homeostasis of intestinal microbiota depends on complex species-species interactions within the bacterial community (17). Physiological benefits were observed to be associated with probiotics consumption in human and animal model without significant effects on microbial composition, indicating that probiotics promoted the homeostasis of intestinal microbiota rather than altered its composition (18, 19). Trillions of bacteria, residing predominantly in the gastrointestinal tract, interact with each other to accomplish systems functions through the flow of energy, matter, and information (20). Microbes in micro-ecological ecosystem depend on interspecific interactions to form a dynamic ecological network, in which species perform different topological roles due to their ecological niche (20). Previous evidence indicated that *B. cereus* G19 could affect microbial interactions and increase the number of generalists to improve the intestinal microbiota homeostasis (21). The symbiotic microbes in the intestine are verified to play a crucially important role in the host metabolism, since microbes possess 100-fold genes more than host and can synthesize a large number of enzymes (22). Therefore, intestinal microbiota is considered to be an auxiliary metabolic organ and participate in metabolic process of host, such as amino acid, carbohydrate, energy, and lipid metabolism through provision of fermentation end products (23–26). Relying on complex species-species interactions, the bacterial community maintains its stability in the intestine, and also accomplishes a system metabolic function simultaneously (27, 28).

Pengze crucian carp (*Carassius auratus* var. Pengze) is a widely farmed freshwater omnivorous fish in China. Previous studies of crucian carp confirmed that high plant protein diets were damaged to the intestinal health (29, 30), while probiotics Bacillus could recover the negative effects and improve growth, antioxidant capability and filet quality (31–34). The studies about Pengze crucian carp have documented that *B. cereus* (35, 36) and *B. subtilis* (37) could improve the growth performance and intestinal health status, while the two probiotics on intestinal microbiota was unclear. And thus, the objective of the current study was to investigate the effects of *B. cereus* and *B. subtilis* on the intestinal microbiota, and further explore their underlying mechanisms of modulating the intestinal microbiota homeostasis.

## Materials and methods

### Experimental procedure and sample collection

Juvenile Pengze crucian carp (*Carassius auratus* var. Pengze) were bought from Fishery Science Research Institute, Jiujiang. Prior to the experiment, the fish were reared in a floating cage (4.0 × 4.0 × 2.0 m) and fed basal diets for 3 weeks to acclimatize to the experiment conditions. After being fasted for 24 h, 180 similar-sized individuals (mean initial weight 12.91 ± 0.02 g) were randomly distributed into nine cages (1.5 × 1.5 × 1.5 m). Each group has three replicates with a density of 20 fish per cage. The dietary ingredient preparation, weighed accurately and mixed thoroughly, basal diets (Control group) made well and dried, were followed by the standard procedure of diets made for the Pengze crucian carp (36, 37). And then, abundant fresh cells of the *Bacillus cereus* (CD group) and *B. subtilis* (BS group) obtained through spreading cultivation, and mixed thoroughly with the basal diets at the dose of 1 × 10^9^ CFU/kg by spraying. The dose of two probiotics was chosen in accordance with previous studies (36, 37), and the concentration of probiotics *B. cereus and B. subtilis* was determine by countess II automated cell counter (Thermo Fisher Scientific, Shanghai, China) before mixing. Fish were fed to apparent satiation three times a day (8: 00, 13: 00, and 16: 00) for 70 days. During the experimental period, water quality conditions were stable (water temperature, 25.5 ± 3.2°C; dissolved oxygen > 6.0 mg L^−1^; NH4^+^-N <0.3 mg L^−1^; ${\text{NO}}_{2}^{-}$-N <0.1 mg L^−1^, respectively). At the end of the experiment, prior to sampling, experimental fish were anesthetized with 100 mg/L MS222 (Tricaine methanesulfonate, Sigma-Aldrich Co. LLC.). The intestinal content in hindgut from eight Pengze crucian carp in each group was collected and frozen at −80°C until further analysis. The experimental protocols together with Pengze crucian carp handling and sampling have been approved by the Committee on Research Ethics of the Department of Laboratory Animal Science, Jiangxi Agricultural University.

### Illumina sequencing of bacterial 16S rRNA gene

PowerFecal™ DNA Isolation Kit (MoBio Laboratories, Inc.) was used for DNA extraction of digesta samples. Amplification of the 16S rRNA V3-V4 region was performed as described previously with barcoded fusion primers of 341F and 805R (38). High-throughput sequencing was performed using the Illumina HiSeq platform. All of the sequencing data can be found in the Sequence Read Archive (SRA) database at NCBI under accession number PRJNA872491.

### Bioinformatics and statistical analysis

The raw sequences were sorted into different samples according to the barcodes by using the BIPES pipeline, followed by a quality-control step remove low-quality amplicon sequences by VSEARCH (39). The clean sequences were then clustered into operational taxonomic units (OTUs) with 99% sequence similarity and annotated using the Ribosomal Database (rdp_16s_v16_sp). A total of 3,056,535 effective sequences and 1,375 OTUs were generated. Alpha diversity and the relative abundance of taxa analyses were calculated by R software v 4.1.3. The Wilcoxon test was used to test the α-diversity index, and the relative abundance of taxa using R software. Principal coordinates analysis (PCoA) based on the Bray-Curtis dissimilarity analyses was employed to visualize bacterial community structure and the difference in bacterial community was calculated by permutational analysis of variance (PERMANOVA) based on Bray–Curtis distance (40).

Based on the abundance profiles of individual OTUs, molecular ecological network analysis was performed to evaluate species-species interactions within bacterial community (http://ieg2.ou.edu/MENA). Random matrix theory (RMT)-based approach was used for ecological network construction and topological roles identification (41). The network was visualized using Circos and Cytoscape 3.9.0, respectively. Based on modularity property, each network was separated into modules by the fast-greedy modularity optimization. According to values of within-module connectivity (*Zi*) and among module connectivity (*Pi*), the topological roles of different nodes can be categorized into four types: peripherals (*Zi* ≤ 2.5, *Pi* ≤ 0.62), connectors (*Zi* ≤ 2.5, *Pi* > 0.62), module hubs (*Zi* > 2.5, *Pi* ≤ 0.62) and network hubs (*Zi* > 2.5, *Pi* > 0.62).

Functional gene and Kyoto Encyclopedia of Genes and Genomes (KEGG) pathways were predicted using PICRUSt2 software (42) against a Greengenes reference database (Greengenes 13.5). And then, the non-metric multidimensional scaling (NMDS) and analysis of similarity (ANOSIM) were used to evaluate the overall differences in predicted bacterial functional composition related to metabolism based on Bray-Curtis distance at KEGG orthology (KO) level (43). A two-sided Welch's *t*-test was used to identify significant different metabolic pathways in the two groups by software STAMP (44), with *P* < 0.05 considered significant.

## Results

### Diversity and composition of the bacterial community

**Figure 1:**
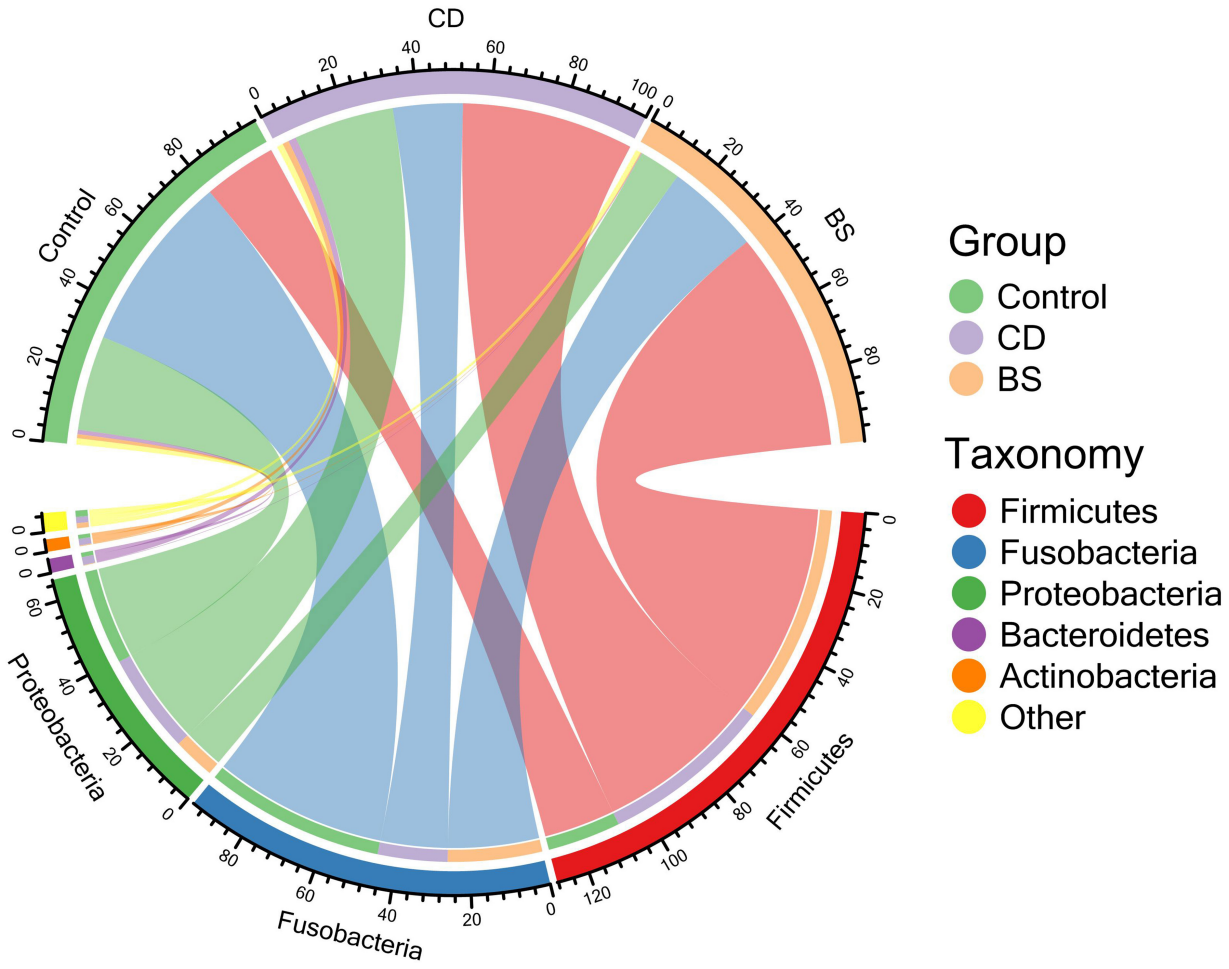
Chord diagram exhibited the relative abundance of bacterial phyla above ≥ a cutoff value of 2%.

**Figure 2:**
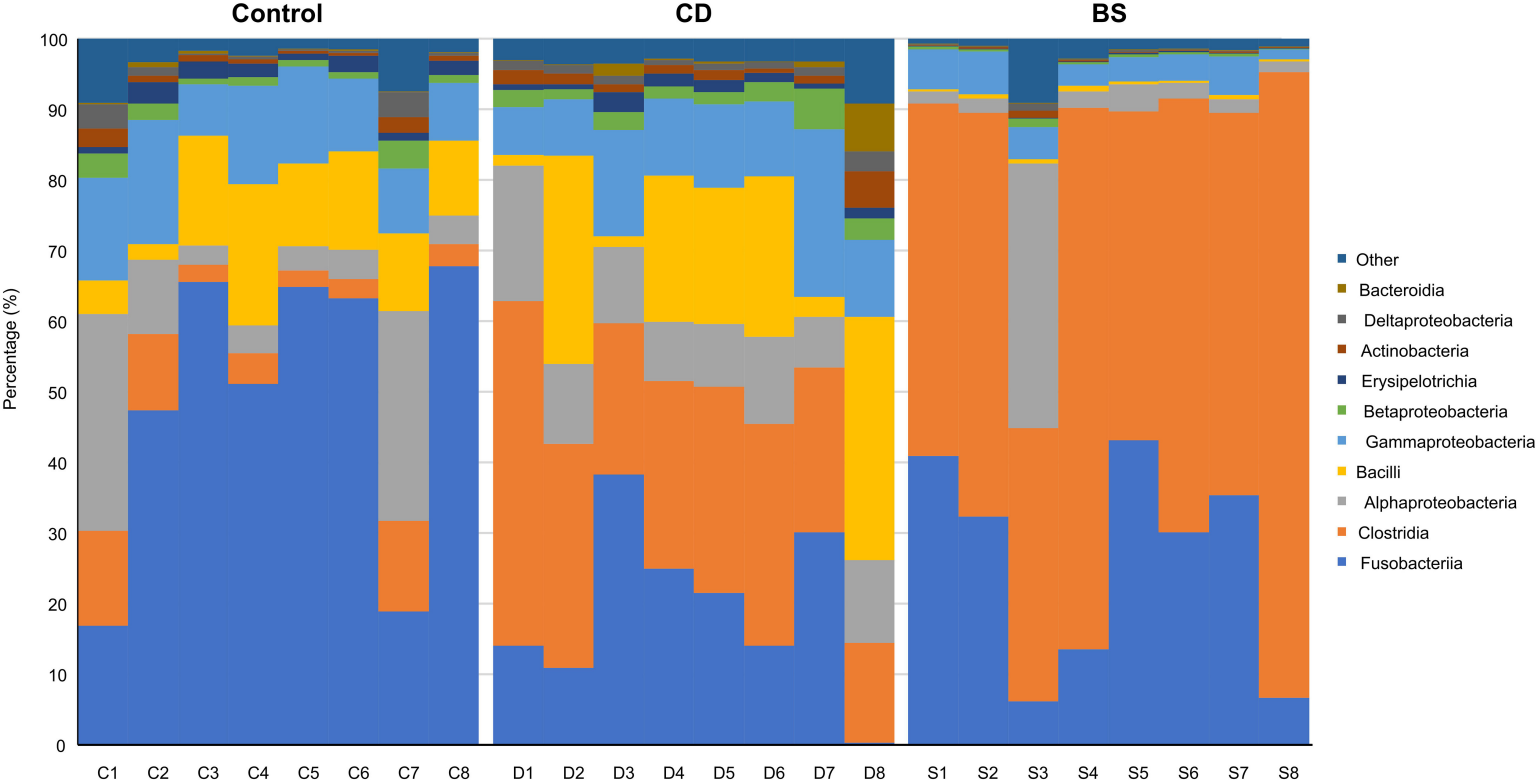
Relative abundances of the top 10 bacterial classes.

Compared to the Control group, dietary supplementation of *B. cereus* increased the number of observed OTUs, Chao1, ACE, Shannon, and Simpson in bacterial community of Pengze crucian carp, whilst a significant difference was recorded in Shannon and Simpson (*P* < 0.05, Table 1). However, no notable difference in α-diversity index mentioned above was observed between BS and Control groups. As shown in Figure 1, the bacterial community of carp was predominated by Fusobacteria (Control: 49.48%; CD: 19.25%; BS: 26.01%), Proteobacteria (Control: 26.16%; CD: 27.63%; BS: 11.60%), and Firmicutes (Control: 20.26%; CD: 47.45%; BS: 60.59%). At class level, fish fed basal diets was mainly rich in Fusobacteriia (49.48%), Gammaproteobacteria (11.86%), Bacilli (11.23%), Alphaproteobacteria (11.15%), and Clostridia (6.50%); Clostridia (28.33%), Fusobacteriia (19.25%), Bacilli (16.58%), Gammaproteobacteria (12.22%), and Alphaproteobacteria (11.23%) took dominate in the intestinal bacterial community in CD group; BS group recorded a high percentage of Clostridia (59.16%), Fusobacteriia (26.01%), Alphaproteobacteria (6.65%), and Gammaproteobacteria (4.15%) in bacterial community (Figure 2).

**Table 1:** The α-diversity index of intestinal microbiota in Pengze crucian carp.

**Parameters**	**Control**	**CD**	**BS**
Obseverd OTUs	559.38 ± 214.66	600.25 ± 109.01	447.5 ± 113.47
Chao1	709.97 ± 242.68	757.75 ± 152.17	650.61 ± 101.68
ACE	714.27 ± 233.94	749.24 ± 147.95	656.66 ± 103.14
Shannon	2.3 ± 0.78^a^	3.3 ± 0.55^b^	1.65 ± 0.78^a^
Simpson	0.69 ± 0.14^a^	0.88 ± 0.04^b^	0.58 ± 0.18^a^

ACE, abundance-based coverage estimator. The values in the same row with different superscripts are significantly different (*P* < 0.05).

**Figure 3:**
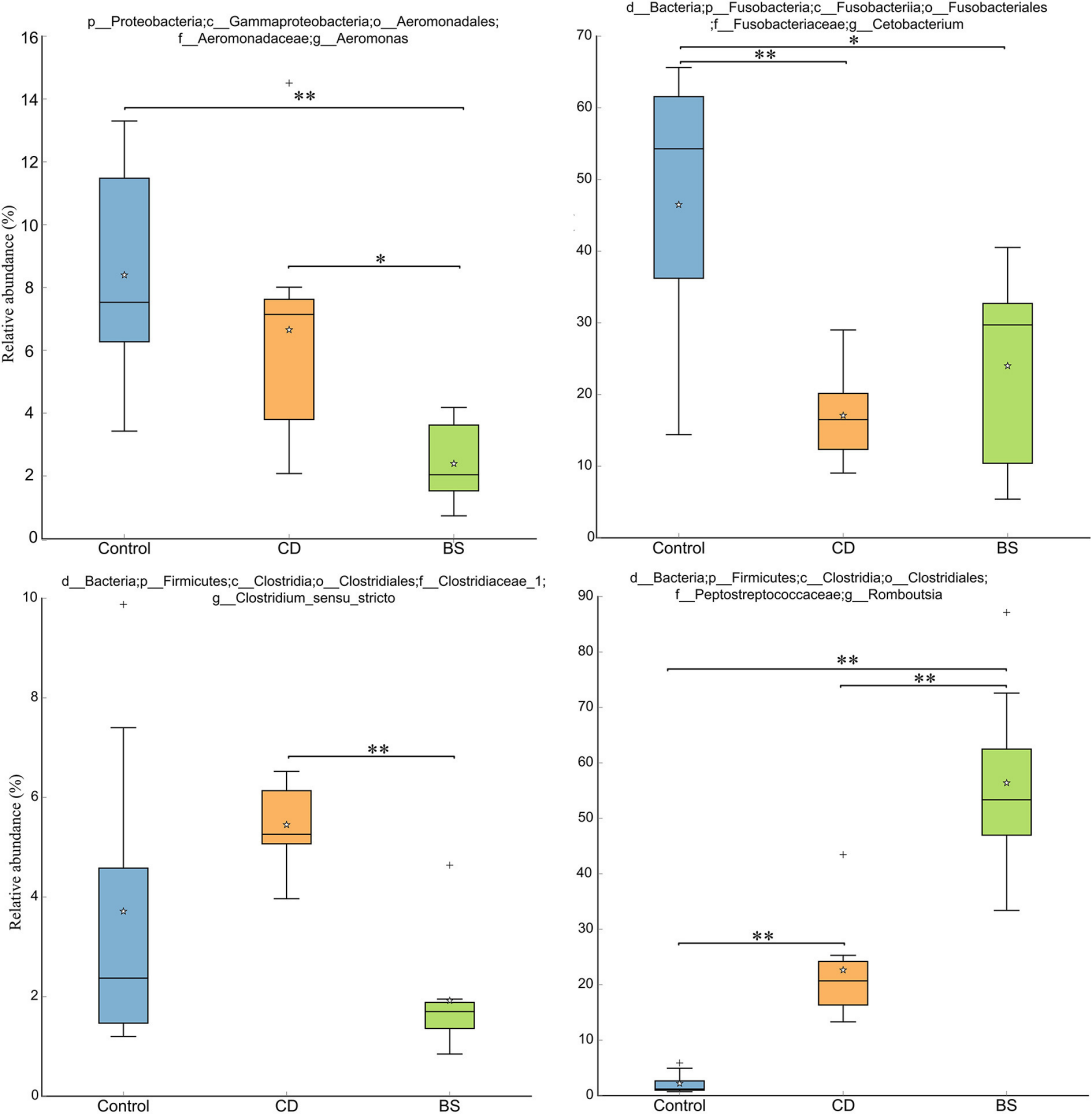
Box plots showing significant variations of relative abundances of intestinal microbiota. **P < 0.01, *P < 0.05.

**Figure 4:**
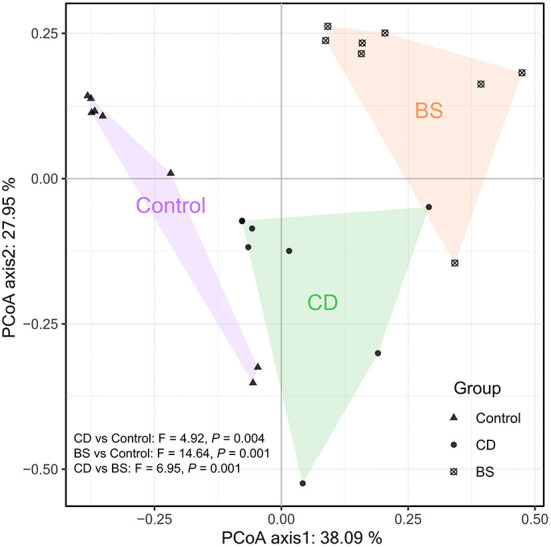
Principal coordinates analysis (PCoA) plot based on Bray-Curtis dissimilarity visualizing dissimilarities in the intestinal bacterial community.

Figure 3 exhibited a significant difference in microbial species among three groups. Fish fed high plant protein diets had a higher average relative abundance of *Aeromonas* (8.39%, *P* < 0.05) and *Cetobacterium* (46.49%, *P* < 0.01) compared to BS group (2.39 and 24.00%, respectively), whilst Control group was higher than CD group (17.08%) in the value for *Cetobacterium* (*P* < 0.01). CD group recorded a higher average relative abundance of *Aeromonas* (6.65%, *P* < 0.05) and *Clostridium_sensu_stricto* (5.45%, *P* < 0.01) compared to BS group (2.39 and 1.92%, respectively). A lowest relative abundance of *Romboutsia* was observed in Control group (2.20%), which was dramatically different from that in CD (22.65%) and BS (56.40%) groups (*P* < 0.01); BS group was higher than CD group (*P* < 0.01) in this respect simultaneously. Strikingly, principal coordinates analysis (PCoA) displayed a clear separation of bacterial community among three groups at OTU level, and significant differences among each other were further confirmed by PERMANOVA (*P* < 0.001, Figure 4).

### Ecological network analysis

**Figure 5:**
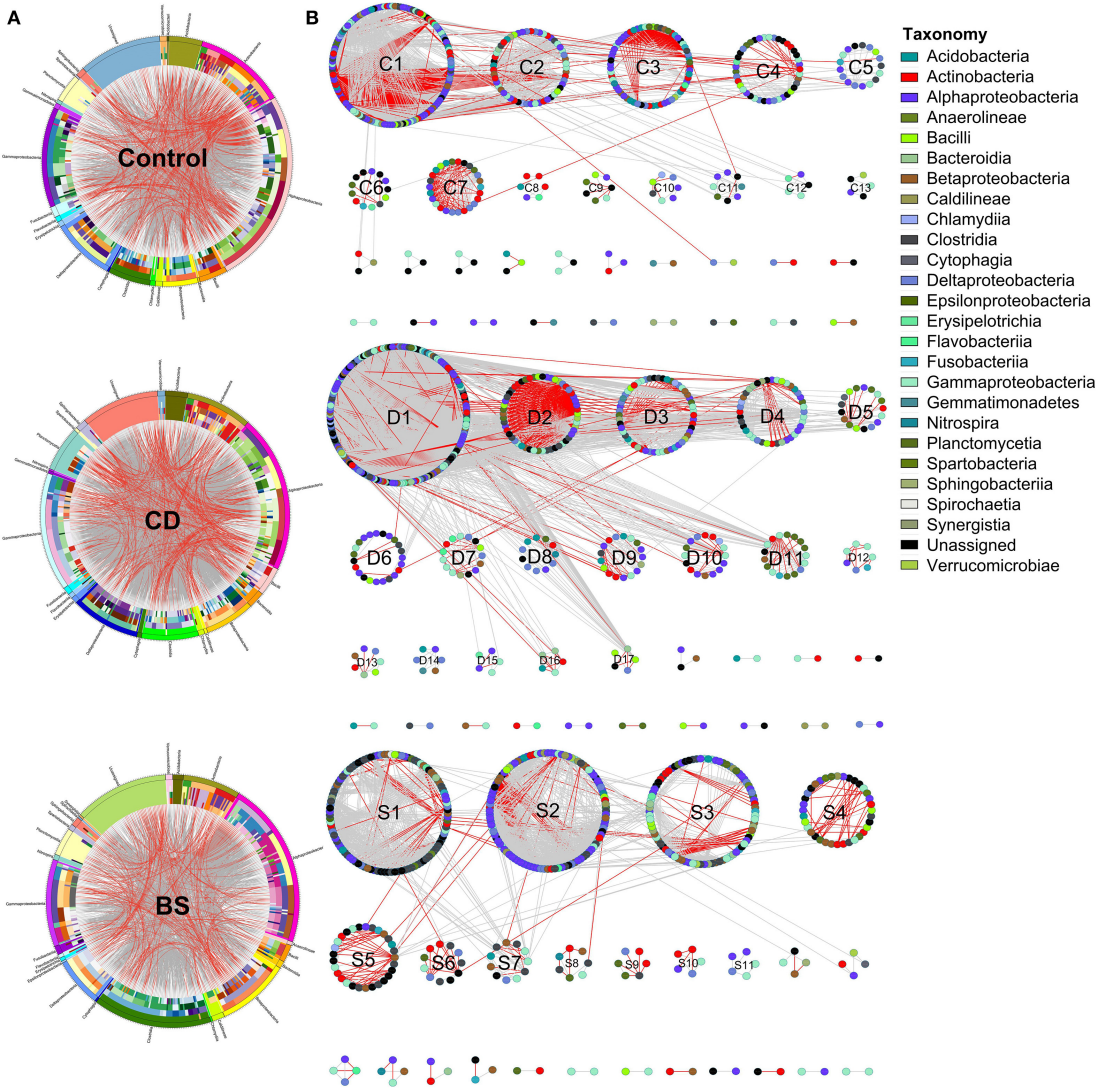
Circular plot **(A)** and ecological network **(B)** descriptions of the interspecific interactions within bacterial community. The width of the bars represents the abundance of each taxon. The bands with different colors demonstrate the source of different genera. The taxonomic levels were class, order, family, genera, and species from the outside to the inside of the circle, respectively. Each node in network graph indicates one OTU. Colors of the nodes indicate different major classes. The edges (gray edge, positive interaction and red edge, negative interaction) inside the circle and ecological network represent the interactions between species.

Circos plot described interactions across different species within bacterial community, and 26 different bacterial classes were observed among three groups (Figure 5A). OTUs from Alphaproteobacteria, Gammaproteobacteria, Clostridia, Actinobacteria, Deltaproteobacteria, Betaproteobacteria, and Planctomycetia were recorded to take dominate in the ecological networks within bacterial community (Table 2). Within Control network, there were 436 OTUs and 2,872 edges including 2,030 gray edges (positive interactions) and 842 red edges (negative interactions) between two OTUs. Total of 509 OTUs and 5,330 edges (gray edges: 4,695; red edges: 635) were observed in CD network, and BS network consisted of 445 OTUs and 2,117 edges (gray edges: 1,766; red edges: 351).

**Table 2:** The composition of the ecological network.

**Index**	**Control**	**CD**	**BS**
Acidobacteria	20	15	6
Actinobacteria	52	44	33
Alphaproteobacteria	112	105	96
Anaerolineae	0	0	2
Bacilli	16	19	10
Bacteroidia	1	11	4
Betaproteobacteria	20	33	42
Caldilineae	4	2	7
Chlamydiia	3	4	1
Clostridia	24	37	68
Cytophagia	1	3	1
Deltaproteobacteria	36	46	28
Epsilonproteobacteria	0	0	1
Erysipelotrichia	3	4	2
Flavobacteriia	3	3	2
Fusobacteriia	6	5	5
Gammaproteobacteria	57	79	50
Gemmatimonadetes	3	2	0
Nitrospira	2	1	3
Planctomycetia	16	30	16
Spartobacteria	1	2	3
Sphingobacteriia	5	7	5
Spirochaetia	0	0	1
Synergistia	0	0	1
Verrucomicrobiae	4	5	4
Unassigned	47	52	54
Total number of OTUs	436	509	445
The number of modules (≥5 OTUs)	13	17	11
The number of module hubs	3	7	8
The number of connectors	3	0	0
The number of gray edges	2,030	4,695	1,766
The number of red edges	842	635	351
Total number of edges	2,872	5,330	2,117

Different separate modules were observed in three ecological networks (Figure 5B). The bacterial ecological network in Control group had 13 submodules (≥ 5 nodes), among which four submodules recorded more than 30 nodes including C1 (141 OTUs), C2 (53 OTUs), C3 (63 OTUs), and C4 (41 OTUs); 17 submodules with more than five nodes were observed in CD network, in which submodules such as D1 (182 OTUs), D2 (54 OTUs), D3 (54 OTUs), and D4 (40 OTUs) were four biggest submodules; BS network possessed 11 submodules (≥5 nodes), among which S1 (141 OTUs), S2 (53 OTUs), S3 (63 OTUs), and S3 (41 OTUs) contained more than 30 nodes. Positive interactions took dominate in these three networks, whereas many red edges were observed between C1 and C2 submodules.

**Figure 6:**
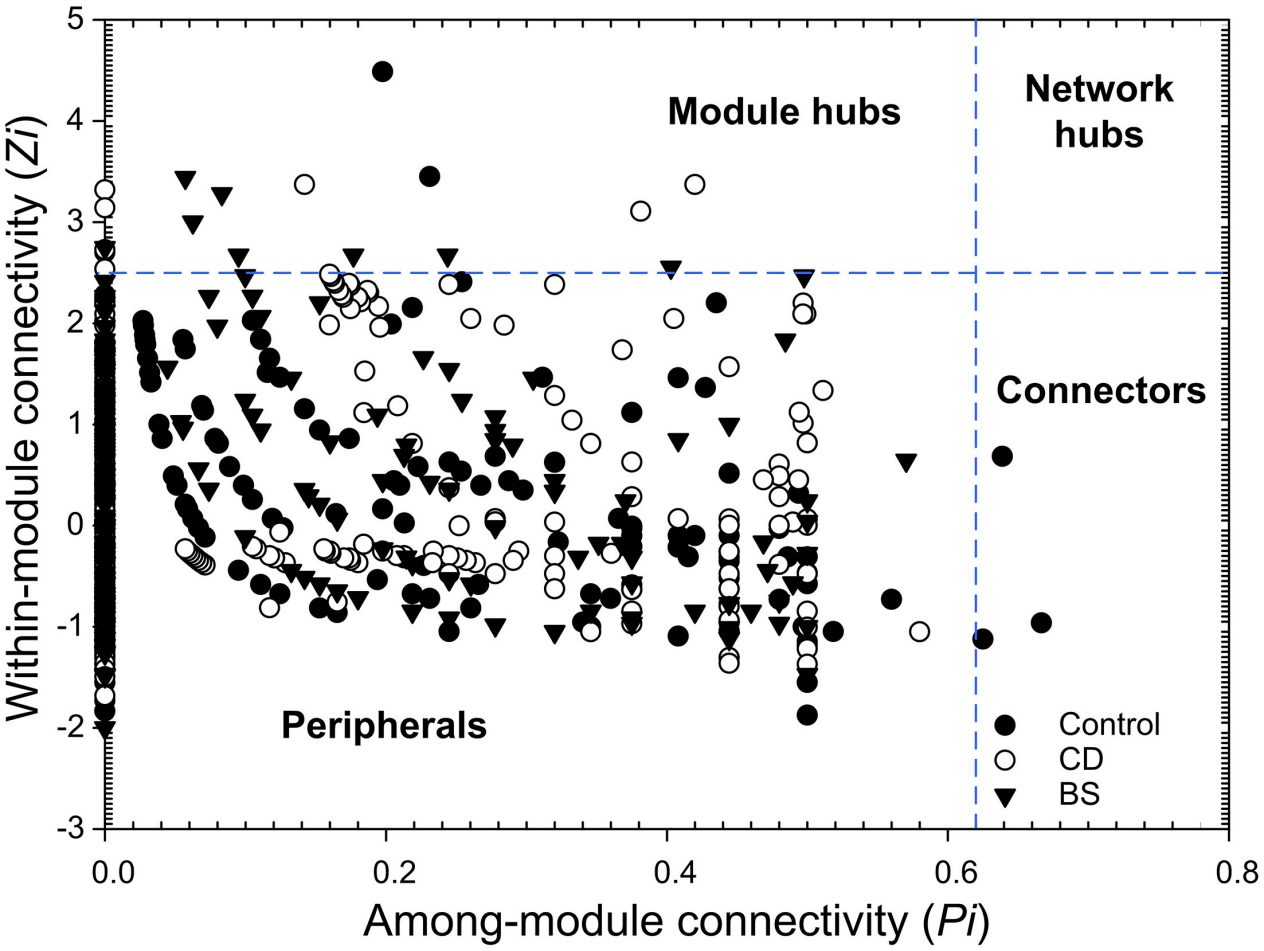
*Z-P* plot showing the topological roles within the ecological network.

The species performed different topological roles in the ecological network, in which most of the nodes were peripherals and several nodes performed as module hubs or connectors (Figure 6). Control network 3 module hubs and 3 connectors mainly from submodules C3 (one OTU from Nitrospira), C4 (three OTUs from Bacilli, Actinobacteria, and Unassigned bacteria), and C5 (two OTUs from Alphaproteobacteria and Gammaproteobacteria); only 7 module hubs were observed in D3 (three OTUs from Actinobacteria, Alphaproteobacteria, and Gammaproteobacteria), D4 (one OTU from Spartobacteria), D6 (one OTU from Alphaproteobacteria), D7 (one OTU from Gammaproteobacteria), D8 (one OTU from Nitrospira) from CD network; similarly, 7 module hubs were found in S1 (two OTUs from Actinobacteria and Clostridia), S3 (four OTUs from Betaproteobacteria, Clostridia, and Sphingobacteriia), S4 (one OTU from Unassigned bacteria), and S6 one OTU from Unassigned bacteria) from BS network (Table 3).

**Table 3:** Topological roles of intestinal microbiota in Pengze crucian carp.

**Treatment**	**Topological roles**	**OTUs**	**Module number**	**Phylogenetic associations**
Control	Module hubs	OTU_225	4	Bacilli
	Module hubs	OTU_582	4	Unassigned
	Module hubs	OTU_369	5	Gammaproteobacteria
	Connectors	OTU_841	3	Nitrospira
	Connectors	OTU_973	4	Actinobacteria
	Connectors	OTU_1346	5	Alphaproteobacteria
CD	Module hubs	OTU_960	3	Actinobacteria
	Module hubs	OTU_1345	3	Alphaproteobacteria
	Module hubs	OTU_1015	4	Spartobacteria
	Module hubs	OTU_202	7	Gammaproteobacteria
	Module hubs	OTU_860	8	Nitrospira
	Module hubs	OTU_1701	6	Alphaproteobacteria
	Module hubs	OTU_41	3	Deltaproteobacteria
BS	Module hubs	OTU_970	1	Actinobacteria
	Module hubs	OTU_702	3	Sphingobacteriia
	Module hubs	OTU_1016	1	Clostridia
	Module hubs	OTU_314	4	Unassigned
	Module hubs	OTU_112	3	Betaproteobacteria
	Module hubs	OTU_1340	3	Clostridia
	Module hubs	OTU_187	3	Betaproteobacteria
	Module hubs	OTU_1173	6	Unassigned

### Functional predictions of intestinal microbiota with PICRUSt2

**Figure 7:**
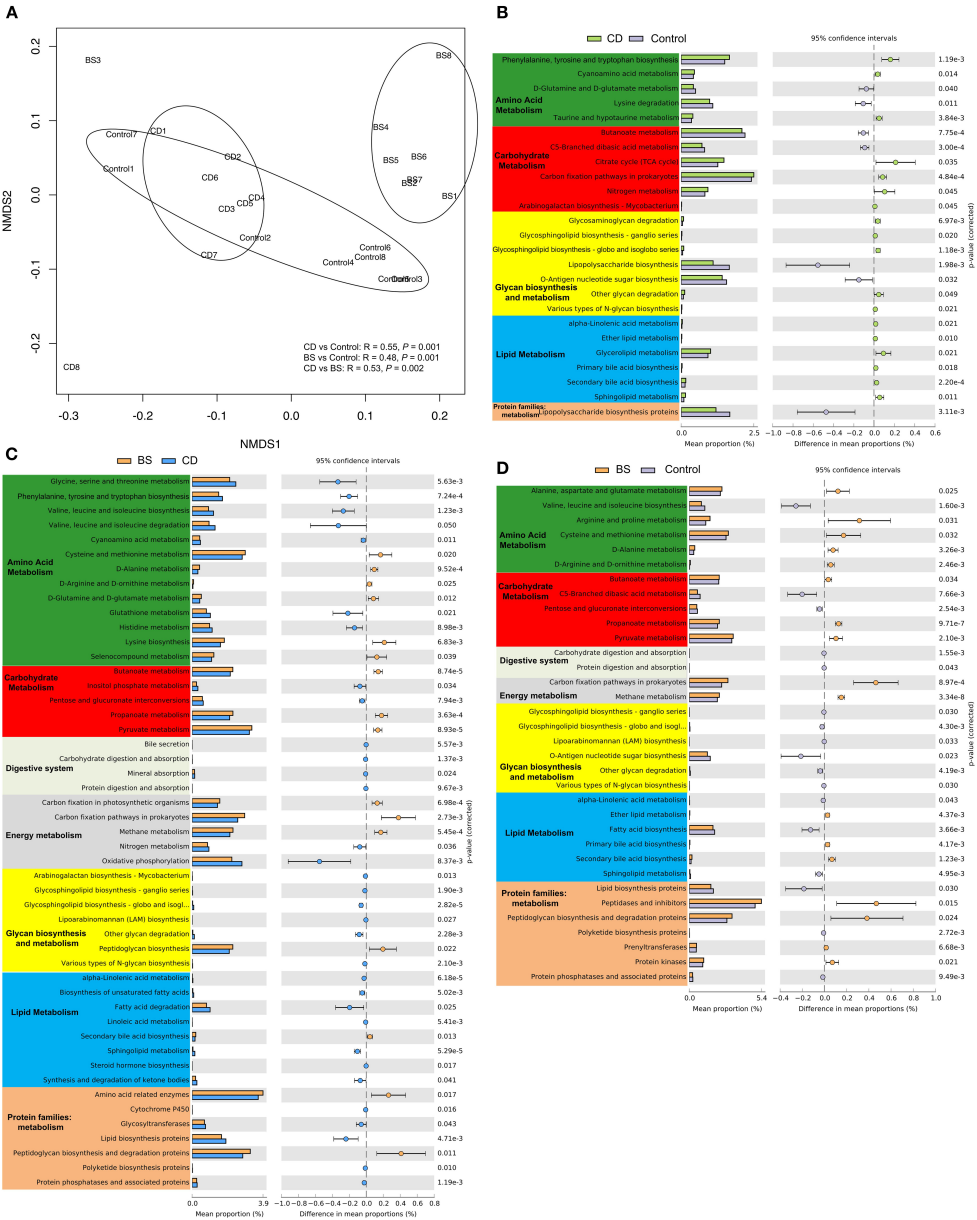
Non-metric multidimensional scaling (NMDS) plot visualizing bacterial functional community dissimilarities using Bray-Curtis distance **(A)**. Differentially abundant KEGG pathways between CD and Control **(B)**, BS and CD **(C)**, or BS and Control **(D)** groups by STAMP.

To understand the bacterial function of the Pengze crucian carp, 7,341 KEGG orthology groups (KOs) were obtained through PICRUSt2 in this study (Figure 7A). The bacterial functional composition was clustered to three groups, and a significant difference between each other was further confirmed through ANOSIM (*P* < 0.05). KEGG functional categories related to metabolic function were further analyzed including Amino acid metabolism, Carbohydrate metabolism, Digestive system, Energy metabolism, Glycan biosynthesis and metabolism, Lipid metabolism, and Protein families: metabolism. There were 24 dramatically different metabolic pathways and one protein family observed between CD and Control groups (*P* < 0.05). Compared to Control group, the bacterial community in CD group possessed 18 enriched metabolic pathways involved in amino acid metabolism (three pathways), carbohydrate metabolism (four pathways), glycan biosynthesis and metabolism (five pathways), and lipid metabolism (six pathways, Figure 7B). Figure 7C exhibited 42 metabolic pathways and seven protein families between CD and BS groups, and microbial function related to amino acid and lipid metabolism was more active in CD group. A total of 27 significantly different metabolic pathways and seven protein families was recorded between BS and Control groups (*P* < 0.05, Figure 7D), and BS group was rich in five pathways from amino acid metabolism, two pathways from carbohydrate metabolism, two pathways from energy metabolism, three pathways from lipid metabolism, and four protein families related to metabolism.

## Discussion

Overuse of plant protein in diets was proved to induce the intestinal disorder together with notably negative effects on the intestinal microbiota of farmed fish (3, 5). Substantial evidences have verified that the alteration in diversity of intestinal bacterial community has been associated with the growth performance of aquatic animal (45), and a reduction in the microbial α-diversity together with a lower growth was found in fish fed with a high soybean meal diet compared to high fish meal diets (5). As we know, the probiotics could improve host health through the modulation of intestinal microbiota. Hence, the objective of this study was to evaluate the function of the two probiotics. The present study indicated that *B. cereus* could dramatically elevate the microbial α-diversity in terms of the increase in Shannon and Simpson index. The positive effect of *B. cereus* on the growth performance of Pengze crucian carp observed in our previous studies (35, 36). Although *B. subtilis* supplementation exhibited no significant influence on the microbial α-diversity, a notable alteration in the microbial composition was observed in Pengze crucian carp, as well in carp fed with *B. cereus*. Firmicutes, Proteobacteria, and Fusobacteria were the dominant phyla colonizing the intestine of farmed fish, and evidences have suggested that high level of plant protein could decrease the abundance of Firmicutes together with an increase in the proportion of Fusobacteria and Proteobacteria in crucian carp (46). Here, Pengze crucian carp fed high plant protein diets also exhibited a high proportion of Fusobacteria and Proteobacteria due to a significant increase of *Aeromonas* and *Cetobacterium*. Specially, *Aeromonas* species from Gammaproteobacteria, gram-negative microbes, are reported as an opportunistic pathogen and have been isolated from wound fish (47, 48). Accordingly, the significant increase in *Aeromonas* indicated the negative effects of high plant protein diets on the intestinal microbiota. However, after consuming probiotics, the abundance of Firmicutes was restored through the increase of *Clostridium_sensu_stricto* and/or *Romboutsia* ratios in this study, confirming the benefits of *B. cereus* and *B. subtilis* on the intestinal microbiota of Pengze crucian carp.

The interspecific interactions enable the intestinal microbiota to form an ecological network, through which the micro-ecological ecosystem can maintain its dynamic homeostasis in host (17). It is well-known that the interspecific interactions present in bacterial community may be due to the species performing similar or complementary function (49). Here, a high cooperation (positive interactions) was shown in bacterial community from middle intestine of Pengze crucian carp fed different diets. According to the ecological theory of *r*/*K* selection, *r*-strategy species are considered to be representative community occupying a nutrient-rich environment, characterized by low competition, high capacity of nutrient utilization, and high growth rates (50). The middle intestine is one place full of various nutrients, creating a nutrient-rich living environment for a *r*-strategy bacterial community in Pengze crucian carp. Previous evidences have revealed that the cooperation-dominated community would be more stable since cooperative interactions are more robust to population perturbations in spatial condition, while competitive interactions, on the other hand, are susceptible to disruption (51, 52). Nevertheless, due to the significant increase in opportunistic pathogen *Aeromonas*, a relatively high competition (negative interactions) was displayed in the intestinal bacterial community of carp fed high plant protein diets. Because of the variation in bacterial community, the carp exhibited characteristic submodules in network, in which the dominant microbiome was the major component. From the ecological viewpoint, peripherals may represent specialists, whereas connectors and module hubs may be related to generalists, and network hubs are super-generalists (53). The generalists played by species act as structural and functional keystone and play a crucial role in maintaining the property of network, so increasing the number of connectors and module hubs can promote the network's stability. Here, the number of connectors and module hubs was observed in carp fed probiotics diets, and similar results was observed in sea cucumber (21). Took together, probiotics *B. cereus and B. subtilis* could improve intestinal microbiota homeostasis of Pengze crucian carp by enhancing the cooperation within bacterial community and increasing the number of generalists in ecological network.

The microbial fermentation is one of most important capacities of intestinal microbiota to participate in host metabolism by secreting digestive enzymes, which are observed to vary among microbial species (20, 22, 26). Therefore, the alteration in the microbial composition caused a notable variation in the microbial metabolic function in the intestine of carp in present study. Accordingly, high plant protein diets disturbed the stability of intestinal microbiota and induced microbial dysfunctions, contributing to the inhibition in the growth performance of Pengze crucian carp (29, 35, 36). Meanwhile, due to the increase of opportunistic pathogen *Aeromonas*, the microbial function related to lipopolysaccharide biosynthesis was dramatically increased in carp fed high plant protein diets, and this was maybe one important reason for the occurrence of intestinal inflammation induced by plant protein since lipopolysaccharide could trigger TLR4-mediated inflammatory pathway (54). As expected, probiotics *B. cereus and B. subtilis* could restore the microbial function, and affect host protein metabolism by elevating the amino acid metabolism of microbial community as well in carbohydrate metabolism, due to the significant increase in Clostridia ratio from Firmicutes. Clostridia has been proved to participate in amino acid metabolism and degrade polysaccharides (55–57). Moreover, the current study revealed that dietary two *Bacillus* probiotics could affect the lipid metabolism of carp by promoting the synthesis of primary and secondary bile acids in enteric cavity. It is widely accepted that microbial fermentation processes depended on the consortium of microbial community through microbe–microbe interactions (27, 58), and thus microbial dysbiosis would lead to metabolic dysfunction (59). Hence, dietary high plant protein diets induced microbial dysfunction, and *Bacillus* supplementation diets could improve the homeostasis of intestinal microbiota and recover the microbial function in Pengze crucian carp.

In conclusion, though altering the microbial composition and affecting species-species interactions and microbial topological roles in the ecological network performed by intestinal bacterial community, probiotics *B. cereus and B. subtilis* could recover the microbial dysbiosis and dysfunction induced by high plant protein diets.

## Data availability statement

The data presented in the study are deposited in the NCBI repository, accession number PRJNA872491.

## Ethics statement

The animal study was reviewed and approved by Committee on Research Ethics of the Department of Laboratory Animal Science, Jiangxi Agricultural University.

## Author contributions

MP designed the experiments and supervised the manuscript. JL and PF carried out the animal experiment and sample analysis with the help of XY and wrote the manuscript. VK revised the manuscript. All authors read and approved the final manuscript.

## Funding

This study was supported by Double Thousand Program of Jiangxi Province (2019) and Excellent Youth Cultivation Project of National Natural Science Foundation of China (20202ZDB01010).

## Conflict of interest

Author XY was employed by Shenzhen Aohua Group Co., Ltd., Shenzhen, China. The remaining authors declare that the research was conducted in the absence of any commercial or financial relationships that could be construed as a potential conflict of interest.

## Publisher's note

All claims expressed in this article are solely those of the authors and do not necessarily represent those of their affiliated organizations, or those of the publisher, the editors and the reviewers. Any product that may be evaluated in this article, or claim that may be made by its manufacturer, is not guaranteed or endorsed by the publisher.
